# Cerium oxide nanoparticles-carrying human umbilical cord mesenchymal stem cells counteract oxidative damage and facilitate tendon regeneration

**DOI:** 10.1186/s12951-023-02125-5

**Published:** 2023-10-03

**Authors:** Xunshan Ren, Huangming Zhuang, Yuelong Zhang, Panghu Zhou

**Affiliations:** Department of Orthopedics, Renmin Hospital of Wuhan University, Wuhan, China

**Keywords:** Tendon, Human umbilical cord mesenchymal stem cell, Reactive oxygen species, Cerium oxide

## Abstract

**Background:**

Tendon injuries have a high incidence and limited treatment options. Stem cell transplantation is essential for several medical conditions like tendon injuries. However, high local concentrations of reactive oxygen species (ROS) inhibit the activity of transplanted stem cells and hinder tendon repair. Cerium oxide nanoparticles (CeONPs) have emerged as antioxidant agents with reproducible reducibility.

**Results:**

In this study, we synthesized polyethylene glycol-packed CeONPs (PEG-CeONPs), which were loaded into the human umbilical cord mesenchymal stem cells (hUCMSCs) to counteract oxidative damage. H_2_O_2_ treatment was performed to evaluate the ROS scavenging ability of PEG-CeONPs in hUCMSCs. A rat model of patellar tendon defect was established to assess the effect of PEG-CeONPs-carrying hUCMSCs in vivo. The results showed that PEG-CeONPs exhibited excellent antioxidant activity both inside and outside the hUCMSCs. PEG-CeONPs protect hUCMSCs from senescence and apoptosis under excessive oxidative stress. Transplantation of hUCMSCs loaded with PEG-CeONPs reduced ROS levels in the tendon injury area and facilitated tendon healing. Mechanistically, NFκB activator tumor necrosis factor α and MAPK activator dehydrocrenatine, reversed the therapeutic effect of PEG-CeONPs in hUCMSCs, indicating that PEG-CeONPs act by inhibiting the NFκB and MAPK signaling pathways.

**Conclusions:**

The carriage of the metal antioxidant oxidase PEG-CeONPs maintained the ability of hUCMSCs in the injured area, reduced the ROS levels in the microenvironment, and facilitated tendon regeneration. The data presented herein provide a novel therapeutic strategy for tendon healing and new insights into the use of stem cells for disease treatment.

**Graphical Abstract:**

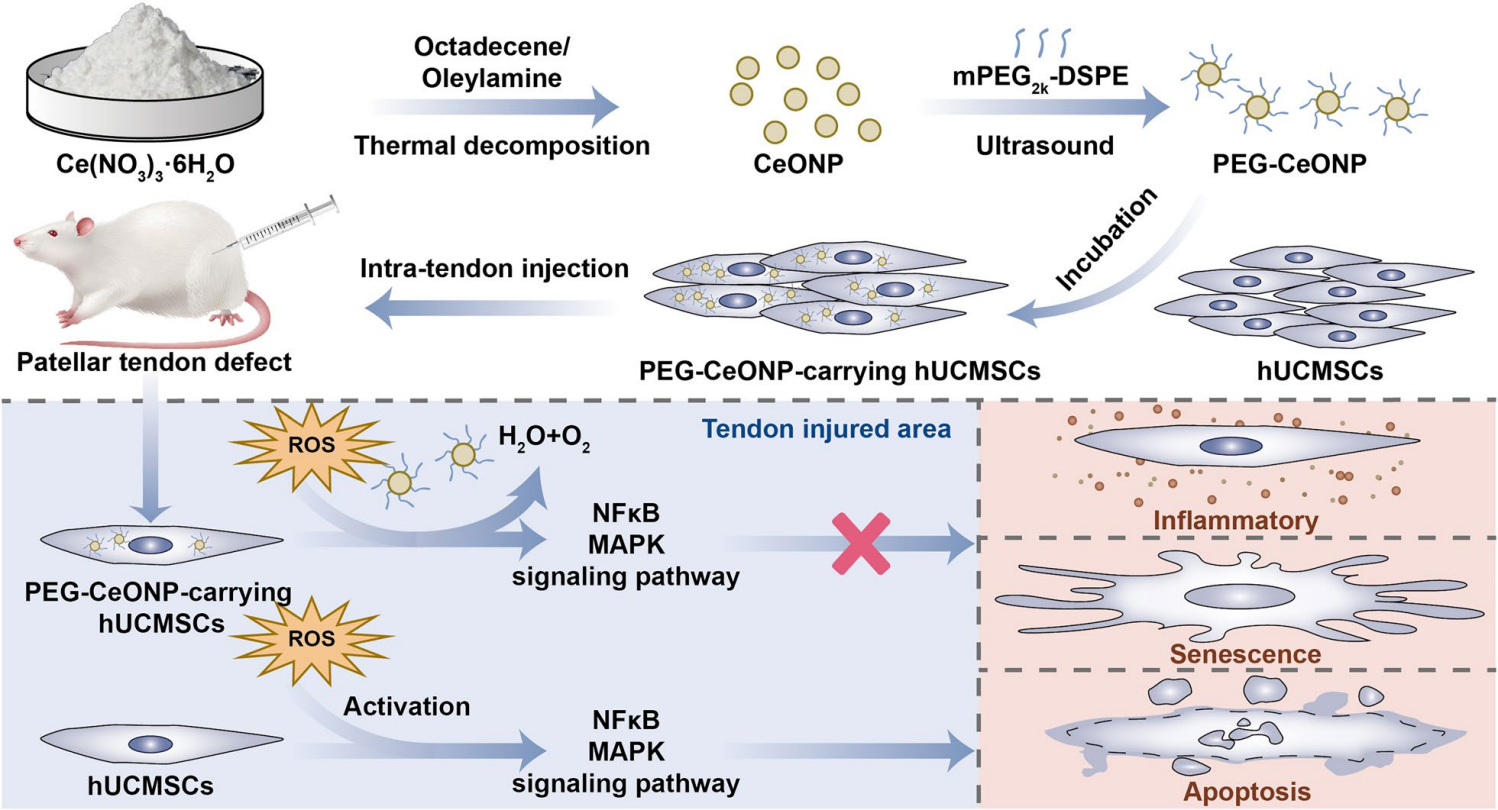

## Background

Tendons are the essential connective tissues that tether skeletal muscles to bones and transmit force. The tendon is easily torn because it is subjected to a high tensile strain. Tendon injuries are among the most common musculoskeletal disorders, affecting more than 32 million people in the United States [1]. Fibrotic scarring is inevitable during healing, resulting in the disruption of tendon matrix continuity and disability [2]. The existing effective treatment options for tendon injuries are limited.

One reason for the inferiority of healed tendons is the limited number of resident cells in the tendon tissues [3–7]. Therefore, cell-based therapies for tendon injury have attracted increasing attention. Because of their self-renewal and differentiation abilities, stem cells are used in multiple tissue regeneration and disease treatments [8–10]. The human umbilical cord is an abundant source of multipotent stem cells, and human umbilical cord mesenchymal stem cells (hUCMSCs) exhibit multiple lineage differentiation potential and immune modulation capacity [11]. hUCMSCs possess many advantages, such as fewer ethical issues, a painless collection process, and a lack of immunity compared to other mesenchymal stem cells (MSCs) [12]. hUCMSCs have general benefits for tissue regeneration in the bone, cartilage, uterus, and brain [13–15]. Jo et al. have reported that hUCMSCs induce rotator cuff tendon regeneration in rat models [16]. Lee et al. observed partial healing in a rabbit model of tendon rupture treated with hUCMSCs [17]. Therefore, hUCMSCs therapy is a potential treatment modality for tendon injuries.

Excessive reactive oxygen species (ROS) are crucial mediators of multiple pathological processes that induce cellular senescence, apoptosis, and dysfunction [18–20]. The therapeutic activity of MSCs is permanently impaired in injured tissues owing to abnormally high oxidative stress [21]. In tendons, both acute and chronic injuries can promote ROS generation, resulting in only a slight improvement in some MSC-related tendon healing studies [1, 22, 23]. Thus, effective antioxidant therapies must be developed in conjunction with stem cell therapies to enhance the efficacy of hUCMSCs.

Cerium is a lanthanide metal element that exhibits repeatable reducibility because of the conversion of cerium ions between trivalent and tetravalent ions. Recently, cerium oxide nanoparticles (CeONPs) have emerged as antioxidants owing to their superoxide dismutase, catalase, and peroxidase activities [24]. Consequently, CeONPs have been widely used in ROS-associated diseases. Du et al. suggested that injection with atorvastatin-loaded CeONPs mitigates acute kidney injury [25]. Lee et al. confirmed that CeONPs can protect against ischemic stroke by scavenging ROS [26]. Similar effects have been demonstrated in liver diseases and glaucoma [27–29]. However, a CeONPs-based defense strategy against oxidative injury during cellular transplantation and tendon injury needs to be developed.

In the present study, we synthesized methyl polyethylene glycol 2000 distearylphosphatidylethanolamine (mPEG_2k_-DSPE)-packed CeNOPs (PEG-CeONPs) and constructed PEG-CeONPs-carrying hUCMSCs. The protective effects of the PEG-CeONPs against oxidative stress were evaluated in vitro. We also assessed the therapeutic effects of the PEG-CeONPs-carrying hUCMSCs in a rat model of tendon injury. This study aimed to develop a novel stem cell modification strategy for tendon injury.

## Methods

### Cell culture

Clinical-grade hUCMSCs were obtained from Wingor Biotechnology Co., Ltd. (Shenzhen, China) and authenticated using cell surface markers and a trilineage differentiation assay. Briefly, 10^7^ hUCMSCs were subjected to analysis for surface markers CD34 (E-AB-F1143D, Elabscience), CD44 (E-AB-F1100D, Elabscience), CD45 (E-AB-F1137D, Elabscience), CD29 (E-AB-F1049D, Elabscience), CD90 (E-AB-F1167D, Elabscience), and CD105 (E-AB-F1310D, Elabscience) by flow cytometry following the manufacturer’s protocol. The lipogenic differentiation ability of hUCMSCs was evaluated using an hUCMSC lipid-induced differentiation medium (PD-019, Procell, Wuhan, China). After lipid droplets appeared in hUCMSCs cultured in lipogenic differentiation medium, they were stained using Oil Red O solution. For the osteogenic differentiation assay, hUCMSCs were cultured in an osteogenic induction differentiation medium (PD-017, Procell, Wuhan, China). After 4 weeks, hUCMSCs were stained with Alizarin Red. The chondrogenic differentiation ability of hUCMSCs was measured using a chondrogenic induction differentiation medium (PD-018, Procell, Wuhan, China) following the manufacturer’s protocol. After 4 weeks, the hUCMSCs cytospheres were fixed, embedded, sliced, and stained with Alcian Blue staining solution and Nuclear Fast Red. Images were captured using an inverted microscope.

### Synthesis and characterization of CeONPs

CeONPs were synthesized using previously reported methods with minor modifications [30]. Briefly, Ce(NO_3_)_3_·6H_2_O (1.736 g, 10294-41-4, Aladdin, Shanghai, China) and oleylamine (3.208 g, 112-90-3, Aladdin) were dispersed in 20 g 1-octadecene (112-88-9, Aladdin) and stirred for 2 h. After heating under a vacuum at 80 °C for 1 h, the mixed solution was heated and maintained at 260 °C for 2 h in an argon atmosphere. CeONPs were washed with cyclohexane and anhydrous ethanol at least three times to collect CeONPs. mPEG_2k_-DSPE (S28722, Yuanye Bio, Shanghai, China) was used to transfer the CeONPs into the aqueous phase through evaporation in an ultrasonic water bath. Finally, the PEG-CeONPs were purified by dialysis (MW cutoff = 8–14 kDa). An inductively coupled plasma optical emission spectrometer was used to calculate the molality of PEG-CeONPs. For cyanine3 (Cy3)-labeled PEG-CeONPs, mPEG_2k_-DSPE was replaced with Cy3-PEG_2k_-DSPE and the above procedure was repeated. The morphology and energy dispersive spectrometer of PEG-CeONPs were detected using an JEM-2100 TEM (JEOL, Tokyo, Japan). FTIR spectroscopy using a FTIR5700 spectrometer (Thermo Electron Corporation, MA, USA). The X-ray photoelectron spectra (XPS) were obtained via an ESCALAB 250 Xi (Thermo Electron Corporation, MA, USA) XPS system.

### Antioxidant capacity of CeONPs

The antioxidant capacity of the CeONPs was assessed using multiple assays according to the manufacturer’s protocol. The free radical scavenging effects of CeONPs were tested using a hydroxyl free radical assay kit (A018-1-1, Nanjingjiancheng, Nanjing, China) and a 2,2-diphenyl-1-picrylhydrazyl (DPPH) free radical scavenging capacity assay kit (A153-1-1, Nanjingjiancheng). Superoxide dismutase (SOD) assay kits (A001-3-2, Nanjingjiancheng) and catalase (CAT) assay kits (A007-1-1, Nanjingjiancheng) were used to evaluate the SOD and CAT enzyme activities of CeONPs, respectively.

### Intervention for hUCMSCs

For constructing the H_2_O_2_-elicited oxidative injury model, hUCMSCs were treated with 500 µM H_2_O_2_ (7722-84-1, Aladdin) for 24 h. hUCMSCs were cultured with 50 µg/mL PEG-CeONPs for 24 h. For pathway activator treatment, hUCMSCs were cultured with 10 ng/mL human recombinant TNFα and 50 µM dehydrocrenatine (DE) for 24 h [31].

### Concentration of cellular ROS

The concentration of cellular ROS was assessed using dichlorodihydrofluorescein diacetate (DCFH-DA, S0033S, Beyotime, Shanghai, China) and dihydroethidium (DHE, 50102ES02, Yeason, Shanghai, China) fluorescent probes following the manufacturer’s protocol. hUCMSCs were washed with PBS and cultured in a serum-free medium supplemented with 10 µM DCFH-DA or 10 µM DHE for 30 min. After washing out the free probe with PBS, images were captured using an inverted fluorescence microscope. The mean fluorescence intensity was measured using ImageJ software (version: 1.8.0).

### Mitochondrial membrane potential

The membrane potential of the isolated mitochondria was measured using the JC-1 Mitochondrial Membrane Potential Assay Kit (G1515, Servicebio) following to the manufacturer’s instructions. After washing with PBS, hUCMSCs were incubated with the JC-1 staining solution at 37 °C for 20 min. The hUCMSCs were then washed three times with JC-1 staining buffer. The images were visualized using an inverted fluorescence microscope and analyzed using ImageJ software.

### Western blotting

Total proteins from hUCMSCs were extracted using RIPA buffer (Servicebio, G2008) supplemented with PMSF (G2008, Servicebio), phosphatase inhibitors (G2007, Servicebio), and 50×cocktail (G2006, Servicebio). Protein concentrations were measured using the BCA protein assay kit (G2026, Servicebio). Protein samples were reduced in SDS sample buffer and separated by 12% SDS-PAGE. After transferring onto PVDF membranes and blocking with 5% skimmed milk, the membranes were incubated with primary antibodies against-β-actin (1:2000, GB11001, Servicebio), BAX (1:2000, 50599-2, Proteintech, Wuhan, China), BCL2 (1:1000, CPA3144, Cohesion, London, UK), P16 (1:1000, A0262, Abclonal, Wuhan, China), P21 (1:1000, A1483, Abclonal), P65 (1:5000, T55034, Abmart, Shanghai, China), P-P65 (1:1000, TP56372, Abmart), IkBα (1:500, TA5002, Abmart), P-IkBα (1:500, TA2002, Abmart), IL-1β (1:1000, A16288, Abclonal), IL-6 (1:1000, CPP1813, Cohesion), TNFα (1:2000, A0277, Abclonal), JNK (1:1000, A0288, Abclonal), P-JNK (1:1000, AP0631, Abclonal), P38 (1:1000, A14401, Abclonal), and P-P38 (1:500, AP0526, Abclonal) overnight at 4 °C. The membranes were washed three times with PBST and incubated with horseradish peroxidase (HRP)-conjugated secondary antibodies (1:5000, GB23303, Servicebio) for 1 h. The ECL substrate (SQ101, Epizyme Biotech, Shanghai, China) was used to visualize the protein bands on a ChemiDoc Touch (Bio-Rad, Hercules, CA, America), and the semi-quantitative analysis of images was conducted through ImageJ software.

### Annexin V/propidium iodide (PI) staining

The percentage of apoptotic hUCMSCs was assessed by Annexin V-fluorescein isothiocyanate (FITC)/propidium iodide (PI) staining (40302ES20, Yeason). Briefly, the hUCMSCs were collected and incubated with 5 µL Annexin V-FITC and 10 µL PI staining solution for 15 min, following the manufacturer’s protocol. Apoptosis rates were evaluated by flow cytometry (Beckman Coulter, USA).

### Colony formation assay

hUCMSCs or PEG-CeONPs-carrying hUCMSCs were exposed to H_2_O_2_ and trypsinized. Five hundred hUCMSCs were replanted in six-well plates and cultured for 3 weeks. After fixing with 4% paraformaldehyde, the plates were stained with a crystal violet solution. ImageJ software was used to test the colony expansion ability.

### Immunofluorescence staining

hUCMSCs were fixed using a 2% paraformaldehyde solution for 15 min, followed by permeabilization using 0.5% Triton X-100 for 20 min at room temperature. The hUCMSCs were washed with PBS and blocked with 5% goat serum. After incubating with primary antibody Ki67 (1:50, A11390, Abclonal) at 4 °C overnight and fluorescent secondary antibody (1:250 GB21303, Servicebio) at 37 °C for 1 h, cells were stained with DAPI, and the images were analyzed under a fluorescent microscope.

### Real-time quantitative PCR (RT-qPCR)

Briefly, 1000 ng of RNA was isolated using RNA isolation kits (R0032, Beyotime) and reverse transcribed using a double-stranded cDNA synthesis kit (G3331, Servicebio). The mixture incubation conditions were: 5 min at 25 °C, 55 °C for 15 min, and 85 °C for 5 s. RT-qPCR was performed in triplicate using the Universal SYBR green fast qPCR mix kit (G3320, Servicebio) on LightCycler® 480 Software (Roche, Swiss Confederation). The primer sequences used in this research were as follows: β-actin, forward primer CACCCAGCACAATGAAGATCAAGAT, reverse primer CCAGTTTTTAAATCCTGAGTCAAGC; P16, forward primer CTGCCCAACGCACCGAATAG, reverse primer AGCTCCTCAGCCAGGTCCAC; P21, forward primer ACCACTGGAGGGTGACTTC, reverse primer CGGCGTTTGGAGTGGTAG; IL-1β, forward primer GTGCACGATGCACCTGTACG, reverse primer ACGGGCATGTTTTCTGCTTG; IL-6, forward primer AAGCAGCAAAGAGGCACTGG, reverse primer TGGGTCAGGGGTGGTTATTG; TNFα, forward primer GAACCCCGAGTGACAAGCCT, reverse primer CCCTTGAAGAGGACCTGGGA.

### In vivo study

This study design was approved by the Laboratory Animal Welfare and Ethics Committee of the Renmin Hospital of Wuhan University (Approval No: 20230101 A) and conducted in compliance with the National Research Council’s Guide for the Care and Use of Laboratory Animals. Eight-week-old male Wistar rats were obtained from SiPeiFu Biotechnology Co, Ltd. (Beijing, China) and divided into the four following groups: sham group (n = 6), tendon injury group (n = 6), tendon injury + hUCMSCs group (n = 6), and tendon injury + PEG-CeONP-carrying hUCMSCs group (n = 6). The rats were anesthetized using 5% isoflurane inhalation, and a single defect (1 mm) was created in the patellar tendon. The latter two groups received 10^6^ hUCMSCs or 10^6^ PEG-CeONP-carrying hUCMSCs through intra-tendon injections once a week [32]. After 4 weeks, the rats were sacrificed and the patellar tendon was collected and fixed for further experiments.

### Histological experiments

Embedded tendons were cut into 5 μm sections and stained with hematoxylin-eosin (HE) and Masson’s trichrome reagent. For immunofluorescence experiments, sections were subjected to antigen retrieval and incubated overnight at 4 °C with anti-SCX (1:200, ab58655, Abcam, Cambridge, UK), anti-TNMD (1:200, ABIN872740, Sizhengbai Biotechnology, Suzhou, China), anti-COL1A1 (1:200, A1352, Abclonal), and anti-COL3A1 (1:200, A3795, Abclonal) antibodies, followed by the corresponding secondary antibodies conjugated with FITC or Cy3. Part of the tendon was freshly frozen in optimal cutting temperature compound for DHE staining. Visualization was performed using a fluorescent microscope and quantification using ImageJ software.

### Statistical analysis

All data in this research are shown as mean ± standard deviation. Shapiro–Wilk normality test was used to perform the normality test. For the data with normal distribution, one-way analysis of variance (ANOVA) followed by Bonferroni’s test (multiple groups) was administered. For the data with non-normal distribution, we performed the Kruskal–Wallis H-test, followed by Dunn’s test (multiple groups). For repeated measurements, ANOVA for repeated measurements was administered. *p* < 0.05 was considered a statistically significant difference.

## Result

### Characterization of hUCMSCs

**Fig. 1:**
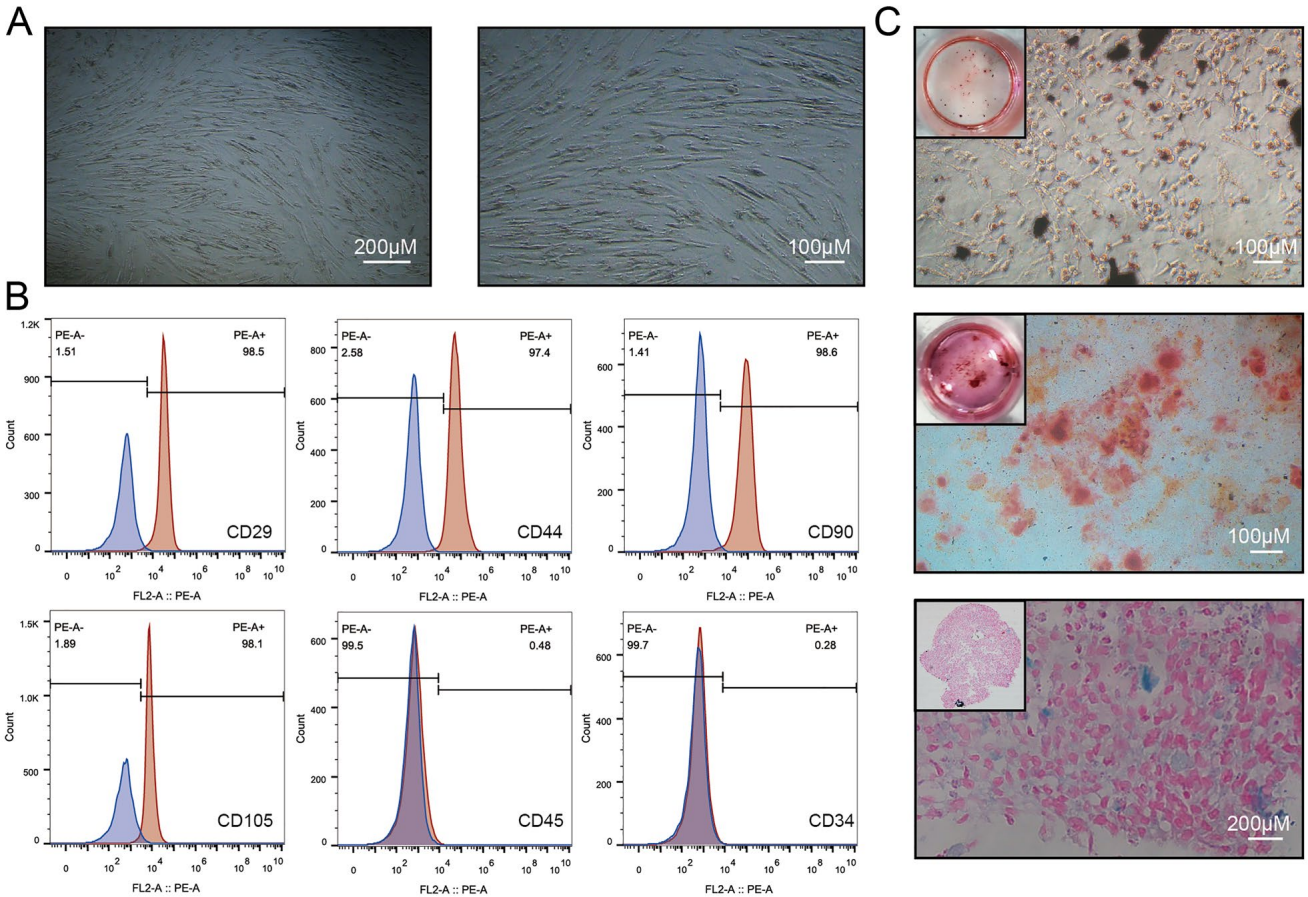
Characterization of hUCMSCs. **A** Primary hUCMSCs were spindle-shaped under light microscopy. **B** Flow cytometry analysis showed that 98.5% of cells expressed CD29, 97.4% expressed CD44, 98.6% of cells expressed CD90, and 98.1% of cells expressed CD105. Meanwhile, only 0.48% and 0.28% expressed CD45 and CD34. **C **Lipid droplets were stained by oil red O after adipogenic differentiation induction of hUCMSCs. Mineralized nodules were stained by Alizarin Red after osteogenic differentiation induction of hUCMSCs. Acid mucopolysaccharide was stained by Alcian Blue after chondrogenic differentiation induction of hUCMSCs

Primary hUCMSCs were identified as described previously [33]. hUCMSCs showed fibroblast-like morphology under a light microscopy (Fig. 1A). Flow cytometry results indicated that hUCMSCs were positive for CD29, CD44, CD90, CD105 and negative for CD45, CD34 (Fig. 1B). Next, hUCMSCs were treated with differentiation media to assess their differentiation potential. Oil red O, Alizarin Red, and Alcian Blue staining showed that the hUCMSCs successfully differentiated into adipocytes, osteocytes and chondrocytes, respectively (Fig. 1C). Thus, hUCMSCs used in this study possessed high purity and excellent differentiation potential.

### Synthesis and antioxidant analysis of PEG-CeONPs

**Fig. 2:**
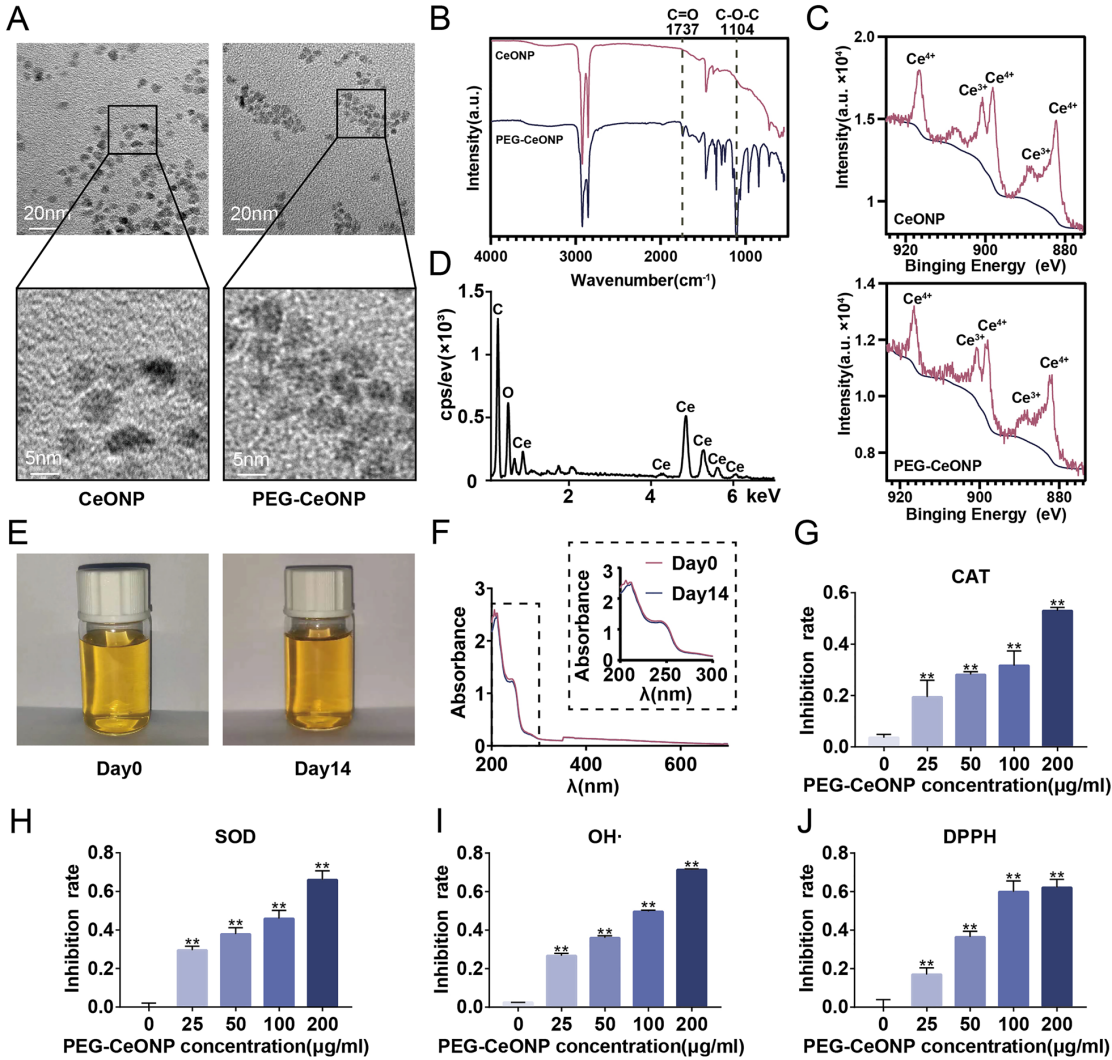
Synthesis and antioxidant analysis of CeONPs. **A** Representative TEM images of CeONPs and PEG-CeONPs. FITR (**B**) and XPS (**C**) analysis of CeONPs and PEG-CeONPs. **D** EDS spectrum of PEG-CeONPs. Appearance (**E**) and UV–visible spectra (**F**) of PEG-CeONPs in aqueous solution for 2 weeks. Inhibition rate of superoxide anion (**G**), hydrogen peroxide (**H**), hydroxyl radicals (**I**), and DPPH (**J**) with different PEG-CeONP concentrations treatment (n = 3). **p < 0.01 versus 0 mg/mL PEG-CeONP

The hydrophobic CeONPs were synthesized by a previously reported thermal decomposition method [30]. After coating with mPEG_2k_-DSPE, CeONPs were transferred to the aqueous phase. As shown in Fig. 2A, the obtained PEG-CeONPs exhibited uniform morphologies with sizes of 5.45 ± 1.08 nm. Compared with hydrophobic CeONPs, there are two additional absorption peaks at 1737 and 1104 cm^−1^, which could be attributed to C=O and C–O–C of DSPE-mPEG respectively [34, 35], indicating the successful surface modified (Fig. 2B). XPS analysis indicates that Ce^3+^ and Ce^4+^ co-exist on the surface of hydrophobic CeONPs and PEG-CeONPs, which provide the chemical basis for the catalytic activities (Fig. 2C) [36]. EDS results revealed a Ce:O atomic ratio of 0.50 (Fig. 2D). Moreover, CeONPs were stable in an aqueous solution for at least 2 weeks, as evidenced by their appearance and the results of the UV-visible spectra analysis (Fig. 2E, F).

The conversion of cerium ions into their trivalent and tetravalent forms typically endows CeONPs with multiple antioxidant enzyme activities [37]. To substantiate this function, we performed a series of in vitro antioxidant assays. The results of SOD and CAT enzyme mimetic activity analysis revealed that PEG-CeONPs inhibited the generation of superoxide anions and hydrogen peroxide in a dose-dependent manner (Fig. 2G, H). DPPH free radical and hydroxyl radicals scavenging assays are widely used to evaluate antioxidant properties [38, 39]. PEG-CeONPs decreased the concentration of free radicals in a dose-dependent manner (Fig. 2I, J). The data suggested that the preparation of PEG-CeONPs with excellent antioxidant properties was successful.

### PEG-CeONPs decreased the concentration of ROS in hUCMSCs

**Fig. 3:**
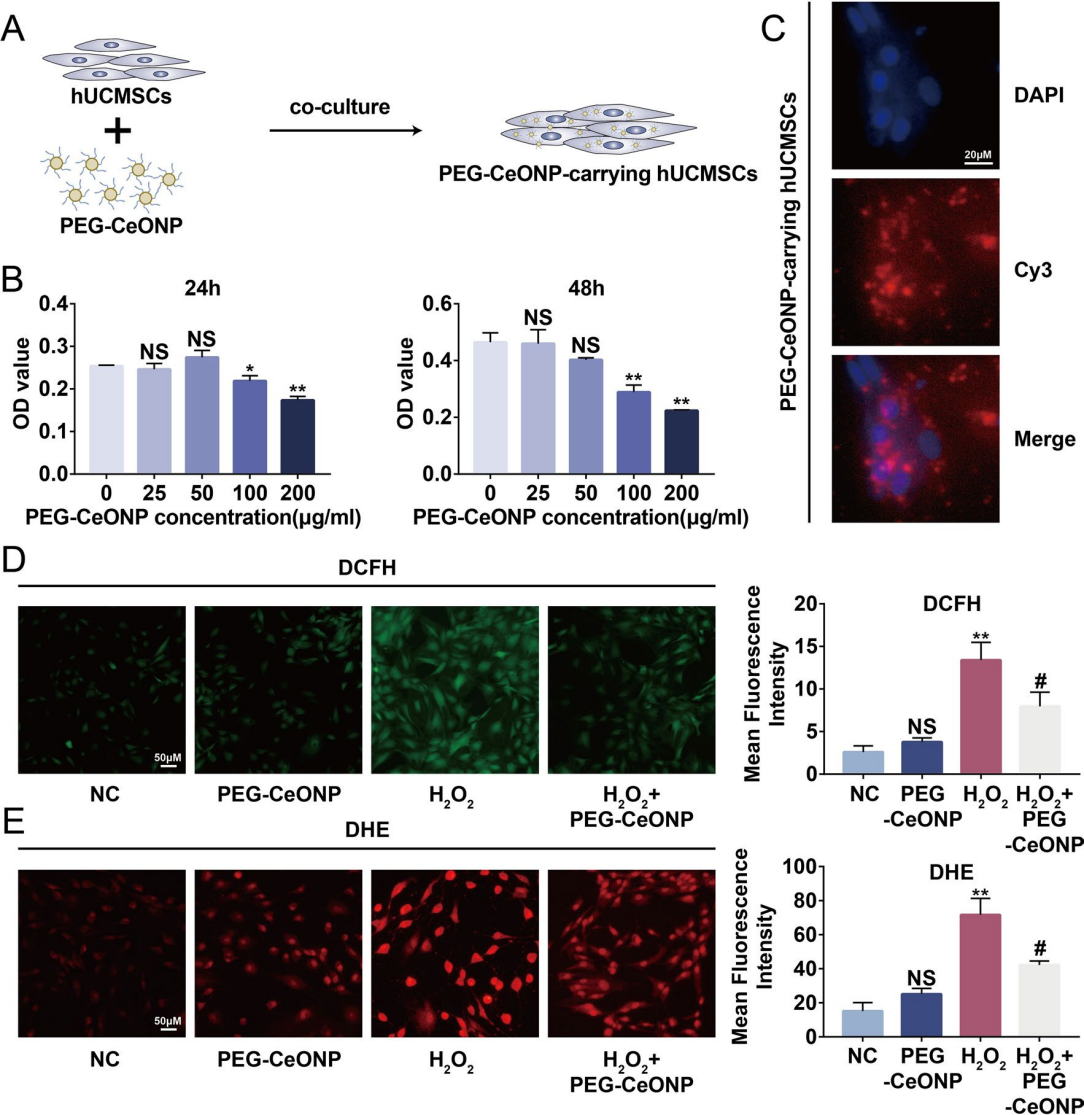
PEG-CeONPs decreased the concentration of ROS in hUCMSCs. **A** A schematic drawing of the hUCMSC carried PEG-CeONPs. **B** hUCMSC viability assessed by the CCK-8 assay after treatment with PEG-CeONPs (n = 3). **C** hUCMSC uptake Cy3-labeled PEG-CeONPs and fluorescence imaging (n = 3). **D**, **E** Representative fluorescence imaging of intracellular ROS evaluated by DCFH and DHE probes and mean fluorescence intensity (n = 3). NS means not significant versus NC or 0 mg/mL PEG-CeONPs, **p < 0.01 versus NC or 0 mg/mL PEG-CeONPs, ^#^p < 0.05 versus H_2_O_2_

We loaded PEG-CeONPs into hUCMSCs to enhance their tolerance to high ROS levels in the injured area (Fig. 3A). CCK8 assay showed that PEG-CeONPs were safe for hUCMSCs with a concentration of less than 50 µg/mL both at 24- and 48-h exposure (Fig. 3B). Thus, we administrated 50 µg/mL PEG-CeONPs to hUCMSCs for 24 h to harvest PEG-CeONPs-carrying hUCMSCs. As shown in Fig. 3C, Cy3-PEG-CeONPs were intaken by hUCMSCs and dispersed around the nucleus. High levels of ROS are characteristic of the injured lesions and are responsible for the limited efficacy of stem cell transplants. Accordingly, hydrogen peroxide was used to mimic the oxidative stress in the injured area of tendon. DCFH-DA and DHE are cell-permeable probes that are widely used to detect the intracellular concentration of H_2_O_2_ or superoxide [40, 41]. DCFH probe imaging suggested that PEG-CeONPs reduced the concentration of ROS after H_2_O_2_ treatment; there was no significant difference between the NC and PEG-CeONPs groups (Fig. 3D). Subsequently, a DHE experiment was conducted and similar results were obtained (Fig. 3E). The results showed that PEG-CeONPs were successfully carried by hUCMSCs and reduced the intracellular ROS levels under H_2_O_2_ intervention.

### PEG-CeONPs-carrying hUCMSCs resisted ROS-mediated apoptosis

**Fig. 4:**
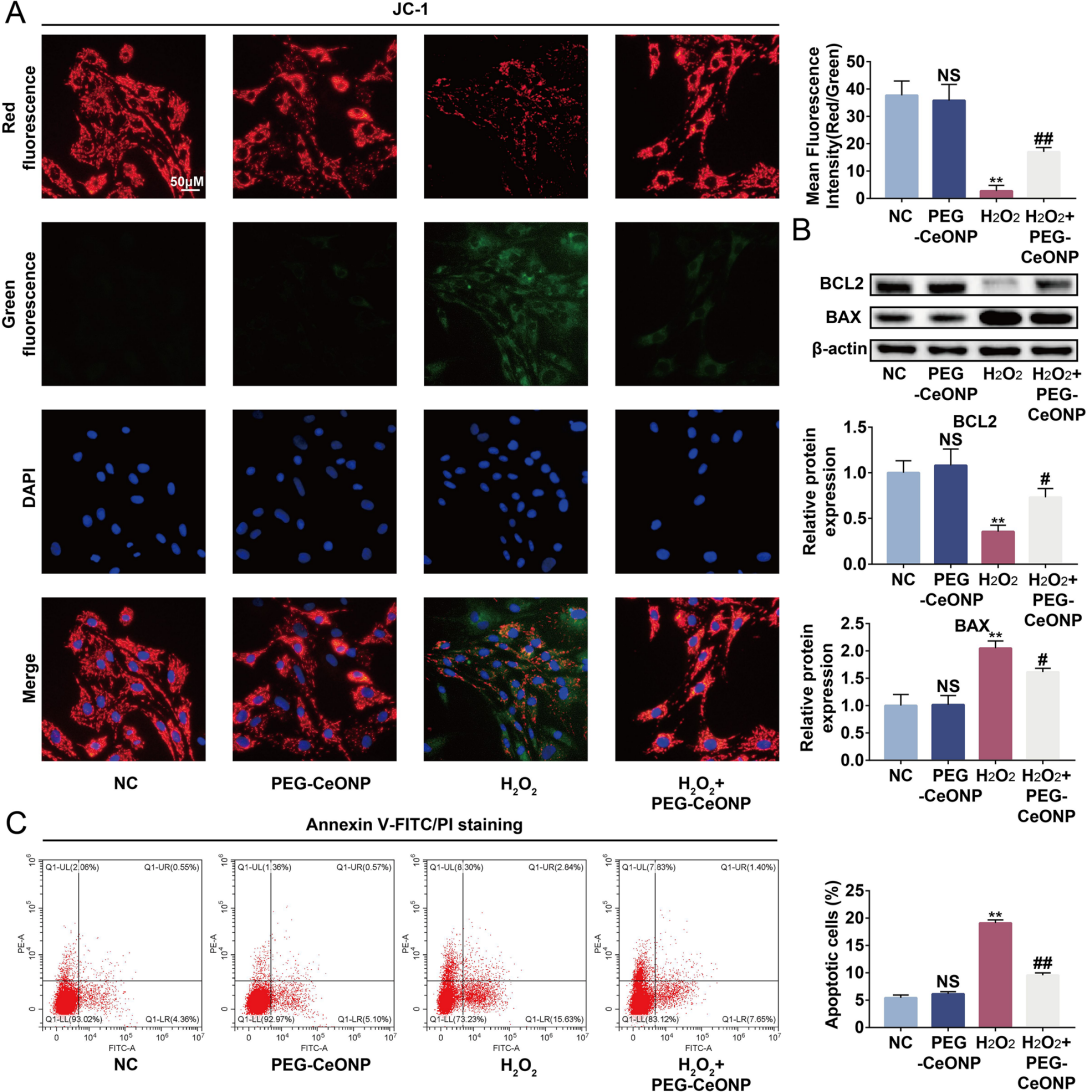
PEG-CeONP-carried hUCMSCs resisted ROS-mediated apoptosis. **A** Representative fluorescence images of mitochondrial potential measured by JC-1 staining and mean fluorescence intensity (n = 3). **B** WB assays and Semi-quantitative analysis of BAX and BCL2 in NC, PEG-CeONPs, H_2_O_2_ and PEG-CeONPs + H_2_O_2_ groups (n = 3). **C** Annexin V/PI staining and cytometry indicated analysis to assess the percentage of apoptotic cells (n = 3). NS means not significant versus NC, **p < 0.01 versus NC, ^#^p < 0.05 versus H_2_O_2_, ^##^p < 0.05 versus H_2_O_2_

High ROS levels promote apoptosis through diverse signaling pathways [42]. Therefore, enhancing the regenerative and anti-apoptotic capacities of stem cells before treatment is considered an interventional strategy for improving therapeutic efficacy [43]. Here, we investigated the effect of PEG-CeONPs on hUCMSCs apoptosis under oxidative stress. Intrinsic cellular apoptosis is always accompanied by loss of mitochondrial membrane potential [43]. We performed a mitochondrial membrane potential assay, and the results showed that healthy polarized mitochondria (JC-1 aggregates, red) decreased and unhealthy depolarized mitochondria (JC-1 monomers, green) increased in the H_2_O_2_ group (Fig. 4A). However, PEG-CeONPs treatment partially reversed this effect. Additionally, PEG-CeONPs treatment reduced the expression of BAX (a pro-apoptotic protein) and increased the expression of BCL2 (an anti-apoptotic protein) under oxidative stress (Fig. 4B). PEG-CeONPs also reduced the percentage of apoptotic hUCMSCs under oxidative stress as shown by the results of flow cytometry (Fig. 4C). These results suggest that PEG-CeONPs-carrying hUCMSCs could resist ROS-mediated apoptosis.

### PEG-CeONPs-carrying hUCMSCs resisted ROS-mediated senescence

**Fig. 5:**
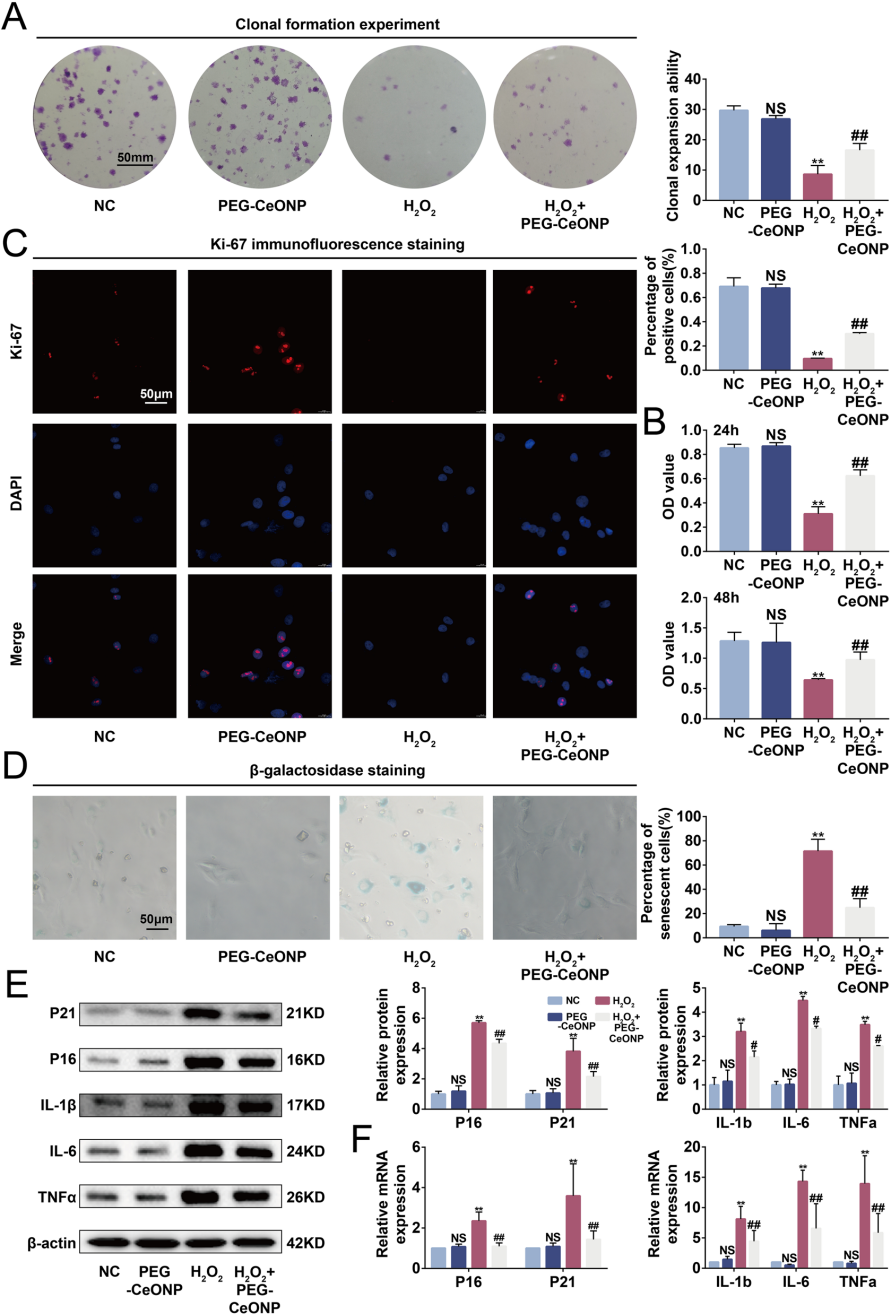
PEG-CeONPs-carried hUCMSCs resisted ROS-mediated senescence. **A** Representative colony formation morphology with result quantification (n = 3). **B** Cell viability was assessed by the CCK-8 assay after treatment with PEG-CeONPs and H_2_O_2_(n = 3).  **C** Ki-67 immunofluorescence staining and percentage of positive cells (n = 3). **D** Representative images of β-galactosidase staining and percentage of senescent cells (n = 3). **E** WB analysis of P21, P16, IL-1β, IL-6 and TNFα in hUCMSC treated with PEG-CeONPs and H_2_O_2_ (n = 3). **F** RT-qPCR analysis of P21, P16, IL-1β, IL-6 and TNFα in hUCMSC treated with PEG-CeONPs and H_2_O_2_ (n = 3). NS means not significant versus NC, **p < 0.01 versus NC, ^#^p < 0.05 versus H_2_O_2_, ^##^p < 0.05 versus H_2_O_2_

Excessive ROS levels disrupt mitochondria and trigger cellular senescence, impairing stem cell function and tissue regeneration [44]. Therefore, we investigated whether PEG-CeONPs exerted their anti-senescent function by scavenging ROS. Irreversible growth arrest is a characteristic of cellular senescence [45]. We performed a clonal formation assay and a CCK-8 assay to evaluate the proliferative capacity. The results showed that PEG-CeONPs counteracted the inhibitory effects of H_2_O_2_ on clonal formation and proliferation (Fig. 5A, B). Moreover, PEG-CeONPs enhanced the expression of Ki-67, a proliferation maker, under oxidative stress (Fig. 5C). Compared with the H_2_O_2_ group, the PEG-CeONPs + H_2_O_2_ group showed decreased senescence, as revealed by the reduced level of β-galactosidase, and decreased expression of the senescence-related factors, including P16 and P21, at the mRNA and protein levels (Fig. 5D−F). Senescent cells secrete pro-inflammatory factors and proteases to alter the tissue microenvironment, which is collectively termed the senescence-associated secretory phenotype (SASP) [46]. RT-qPCR and Western blot results indicated that treatment with PEG-CeONPs decreased the expression of SASP makers, including IL-1β, IL-6, and TNFα, which were enhanced by H_2_O_2_ (Fig. 5E and F). In summary, PEG-CeONPs-carrying hUCMSCs counteracted senescence induced by high ROS levels.

### PEG-CeONPs-carrying hUCMSCs resisted apoptosis and senescence through NFκB and MAPK signaling pathways

**Fig. 6:**
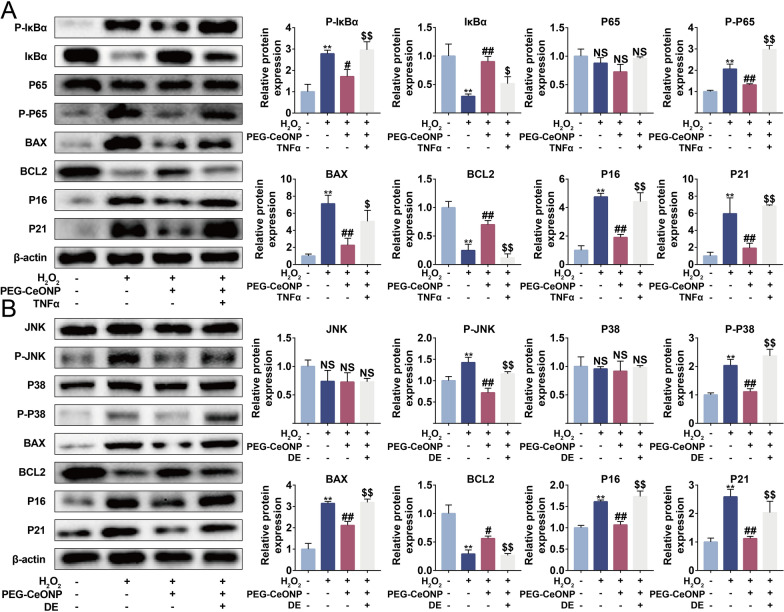
PEG-CeONPs-carried hUCMSCs resisted to apoptosis and senescence through NFκB and MAPK signaling pathway. **A** WB analysis of IκBα, P-IκBα, P65, P-P65, BAX, BCL2, P16 and P21 in hUCMSC treated with PEG-CeONPs, H_2_O_2_ and TNFα (n = 3). **B** WB analysis of JNK, P-JNK, P38, P-P38, BAX, BCL2, P16 and P21 in hUCMSC treated with PEG-CeONPs, H_2_O_2_ and DE (n = 3). **p < 0.01 versus NC, ^#^p < 0.05 versus H_2_O_2_, ^##^p < 0.05 versus H_2_O_2_, ^$^p < 0.05 versus PEG-CeONPs, ^$$^p < 0.01 versus PEG-CeONPs. NS means not significant

Given that nuclear factor kappa-B (NFκB) and mitogen-activated protein kinase (MAPK) signaling pathways are involved in the transduction of oxidative signal and cellular apoptosis, we performed further experiments to elucidate the mechanism underlying PEG-CeONPs in hUCMSCs exposed to excessive oxidative stress [47, 48]. As shown in Fig. 6A and B, H_2_O_2_ elevated the expression of P-IκBA, P-P65, P-JNK, and P-P38, and reduced the level of IκBA, indicating the activation of NFκB and MAPK signaling pathways. To investigate the roles of NFκB and MAPK signaling pathways in the anti-apoptosis and anti-senescence effect of PEG-CeONPs, we treated PEG-CeONPs-carrying hUCMSCs with NFκB activator human recombinant TNFα and JNK/P38 activator DE [49, 50]. The application of human recombinant TNFα and DE counteracted the downregulatory effect of PEG-CeONPs on the BAX, P16 and P21 at the protein level and decreased the expression of BCL2. Thus, our data verified that PEG-CeONPs resisted apoptosis and senescence by inactivating the NFκB and MAPK signaling pathways.

### Local administration of PEG-CeONP-hUCMSCs effectively promotes the recovery of patellar tendon defect

**Fig. 7:**
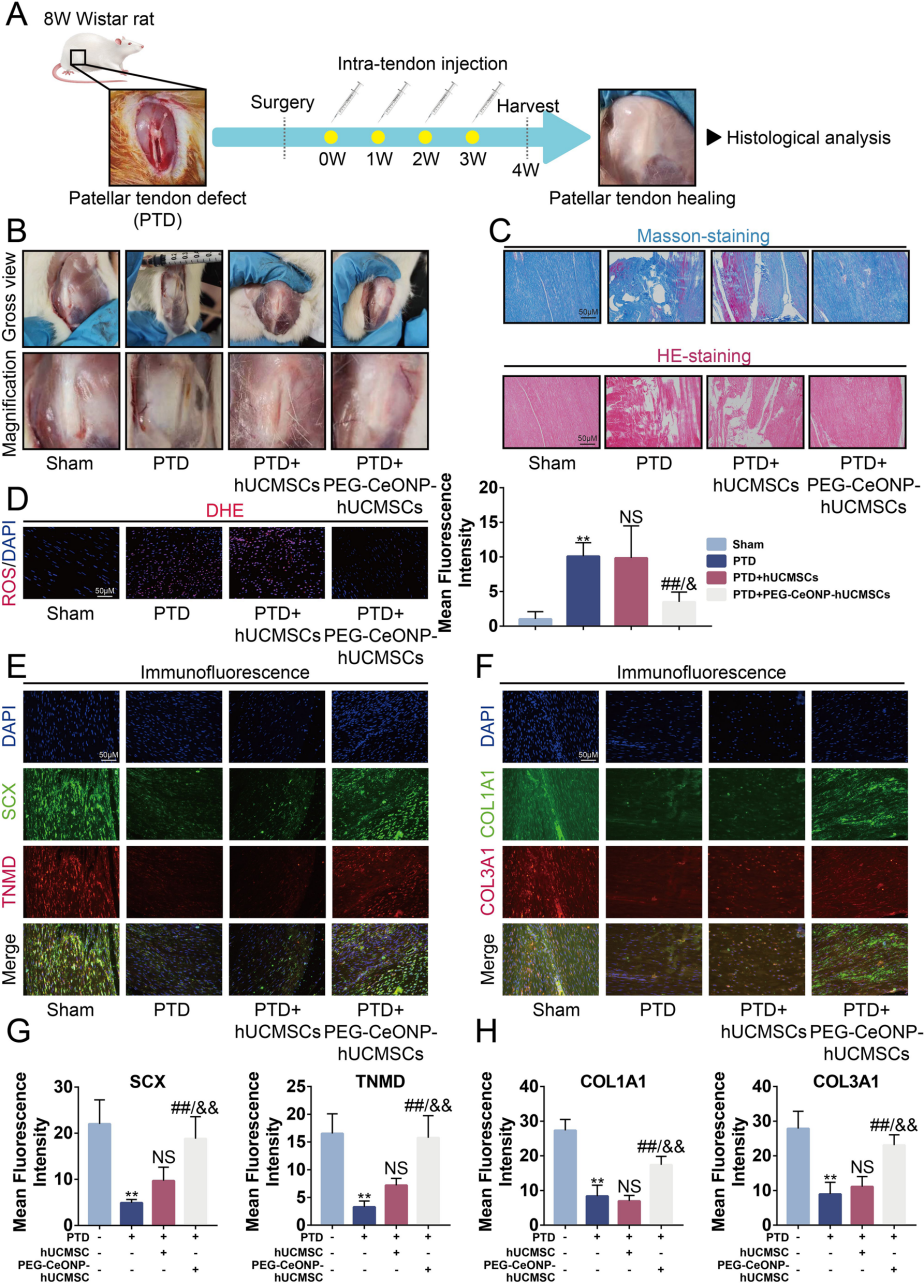
Local administration of PEG-CeONP-hUCMSCs effectively promotes recovery of patellar tendon defection. **A** A schematic drawing of animal experiments. **B** The appearance of tendon-injured areas. **C** Masson and HE staining of the tendon with PTD, PTD + hUCMSCs and PTD + PEG-CeONP-hUCMSCs treatment. **D** Representative fluorescence imaging of intracellular ROS evaluated by DHE probes and mean fluorescence intensity (n = 6). **E**, **F** Representative fluorescence imaging of immunofluorescence of TNMD, SCX, COL3A1 and COL1A1 (n = 6). **G**, **H** mean fluorescence of immunofluorescence image. NS means not significant versus PTD (n = 6), **p < 0.01 versus Sham, ^##^p < 0.01 versus PTD, ^&^p < 0.01 versus PTD + hUCMSCs, ^&&^p < 0.01 versus PTD+ hUCMSCs

High levels of oxidative stress contribute to the limited efficacy of stem cell transplantation in injured areas. Thus, to investigate the advantages of the local injection of PEG-CeONP-hUCMSCs in tendon healing, we established a patellar tendon defect (PTD) model induced by surgery [2]. hUCMSCs and PEG-CeONP-hUCMSCs were injected into the tendon once per week (Fig. 7A). After 4 weeks of treatment, tendon tissues collected from the PTD group presented a significant defect. In contrast, tendons from the hUCMSCs and the PEG-CeONP-hUCMSCs presented smaller defects, especially in the latter (Fig. 7B). HE and Masson staining showed that treatment with PEG-CeONP-hUCMSCs reversed the disorderly arrangement of collagen fibers and attenuated the formation of vacuole-like structures (Fig. 7C). To evaluate the oxidative stress levels, the ROS fluorescent probe DHE was loaded into the tissues. Fluorescent images and semiquantitative analysis showed that PEG-CeONP-hUCMSCs remarkably inhibited the increase in ROS levels evoked by the injury compared to the hUCMSCs intervention (Fig. 7D). Moreover, PEG-CeONP-hUCMSCs enhanced tendon repair as evidenced by the higher expression of tenogenic markers SCX, TNMD, COL1A1, and COL3A1 (Fig. 7E–H). Taken together, the local administration of PEG-CeONP-hUCMSCs effectively promoted the repair of patellar tendon defects in rat models.

## Discussion

Tendon injuries cause prolonged disability with high incidence [51]. The repair of tendon injuries is incomplete because of fibrotic scarring. Plenty of treatment strategies have been developed to enhance tendon healing; however, their efficacy requires improvement. Stem cell transplantation holds great promise in multiple diseases, including tendon injuries, as stem cells secrete a diverse repertoire of growth factors such as vascular endothelial growth factors, fibroblast-like growth factors, and insulin-like growth factors [52–54]. However, the poor survival of the stem cells in injured tissues impedes their therapeutic development, which is attributed to the high ROS concentrations in the injured areas.

Cerium oxide is a stable lanthanide metal element and the pale-yellow oxide form of the most abundant rare-earth metal [55]. Driven by a ground-state Ce 4f electron, the powerful Ce^3+^ to Ce^4+^ redox couple contribute to considerable reducibility [56]. Possessing superoxide dismutase, catalase, and peroxidase activities, CeONPs have been considered antioxidant agents [57–59]. CeONPs, with a higher surface-area-to-volume ratio, have better reducibility than cerium oxide with larger particles because of oxygen vacancies and Ce^3+^ mostly existing on the surface [60]. In our study, ultrasmall PEG-CeONPs with a size of 5.45 nm were synthesized and exhibited significant reducibility. Previous studies have been conducted to enhance the therapeutic efficacy of cell transplantation by pre-treating stem cells with natural drugs such as curcumin, Exendin-4, and resveratrol or by genetically modulating [61–63]. The above options often face high cost, non-specificity, limited activity and consequent uncontrolled side effects and thus fail in clinical trials [28]. In comparison, CeONPs, the artificial enzyme, has the advantage of low cost, high efficiency and stability, massive production, and easy handling, which attracted us to explore its role in cell transplantation [64]. Nanoparticles can be actively taken up by cells via endocytosis, indicating PEG-CeONPs can be carried by hUCMSCs and transplanted to the injured areas [65]. Although generally safe, concentration-dependent cytotoxicity of CeONPs has been observed in various cell types [66, 67]. Therefore, we explored and verified the appropriate loading conditions under which PEG-CeONPs exhibited both biocompatibility and antioxidant capacity in hUCMSCs.

Apoptosis, a form of programmed cell death, markedly affects stem cell transplantation [21]. Oxidative stress can induce apoptosis in many ways, including activation of the mitochondrial pathway (intrinsic) and activation of death receptors at the cell surface (extrinsic) [68]. Apoptotic stem cells possess severely compromised regenerative potential and secret fewer growth factors; therefore, improving the outcome of stem cell therapy is a novel interventional strategy, as exemplified by endowing stem cells with pro-survival and anti-apoptotic genes [43]. In this work, we confirmed that PEG-CeONP-carrying hUCMSCs could resist oxidative damage-induced cellular apoptosis in vivo and in vitro. In addition to causing apoptosis in transplanted cells, high oxidative stress can damage in site tendon-derived stem cells and deteriorate tendon healing [69]. Our study suggests that injection of PEG-CeONP-carrying hUCMSCs remodeled the microenvironment in the injured area by scavenging and inhibiting apoptosis. Senescence is a cellular state characterized by irreversible growth arrest and an altered epigenetic mechanism [70]. Recently, the transplantation of senescent cells into young animals was shown to result in persistent physical dysfunction [71, 72]. Senescence can be triggered by various stressors, such as multiple generations, activation of oncogenes, and ROS-induced DNA damage [73]. The accumulation of senescent cells in tendon tissue is a possible pathogenesis mechanism underlying tendinopathy [74]. Our data revealed that PEG-CeONPs protected hUCMSCs from H_2_O_2_-evoked cellular senescence and decreased the expression of senescence-associated molecules P16 and P21 in rats.

**Fig. 8:**
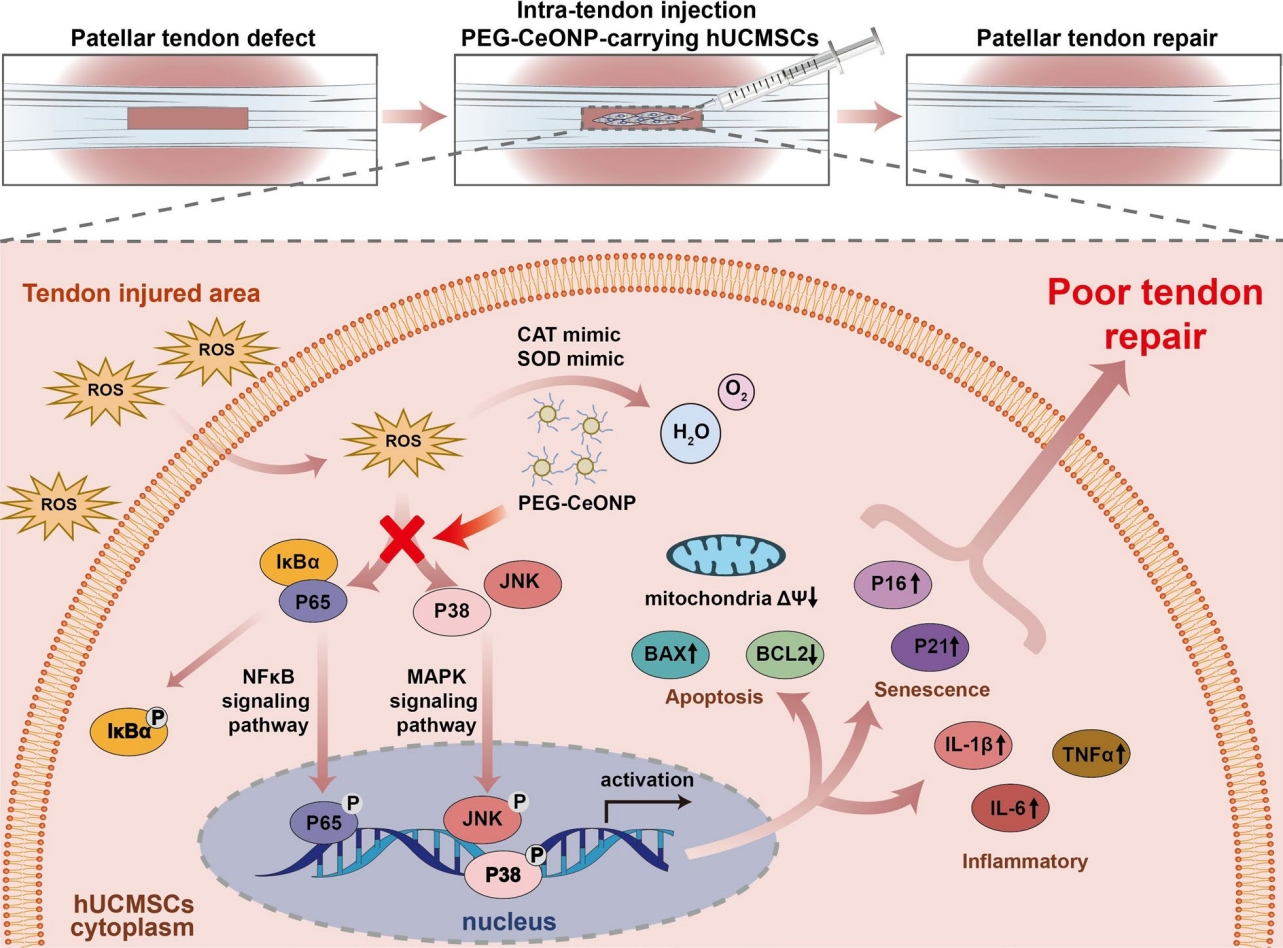
Schematic illustration of PEG-CeONP-carrying hUCMSCs counteract oxidative damage and facilitates tendon regeneration

NFκB is a pleiotropic, redox-sensitive, nuclear transcription factor regulating the expression of many genes and associating with multiple biological processes, including apoptosis and senescence [75, 76]. The NFκB signaling pathway can be activated by H_2_O_2_ at different sites [77]. The activation of the NFκB signaling pathway is involved in musculoskeletal diseases including tendon diseases [78]. The MAPK pathway is a representative stress-responsive signaling pathway that induces cellular responses to divergent environmental stimuli [79]. Increased ROS generation leads to the activation of MAPK cascades, including c-Jun NH2-terminal kinase (JNK), and p38 MAPK [80]. Apoptosis and senescence are mediated by the MAPK signaling pathway [81, 82]. Therefore, we sought to explain the function of PEG-CeONPs by assessing the activation of NFκB and MAPK signaling pathways. Our data showed that PEG-CeONPs inhibited the activation of NFκB and MAPK signaling pathway in H_2_O_2_-treated hUCMSCs, and human recombinant TNFα and DE reversed the effect of PEG-CeONPs loading, indicating that PEG-CeONPs acted through the NFκB and MAPK signaling pathways (Fig. 8).

Various biomaterials strategies have been developed for tendon regeneration because of the poor repairs induced by low cellularity sources [83], poor blood supply [84], inflammatory microenvironment, and excessive oxidative stress [1]. Currently, biomaterials strategies for tendon regeneration mainly include scaffolds to excel at mechanical stabilization and hydrogels to deliver biochemical cues or cells [85]. For instance, A polymeric three-dimensional scaffold was reported to retain tendon-like mechanical properties and accelerate the healing progression [86]. Ren et al. designed a high-tenacity shape-adaptive hydrogel to deliver fibroblast growth factor [87]. Ji et al. proposed a cocktail-like hydrogel to transmit bone marrow mesenchymal stem cells [88]. The main issue of our research would be concerns about is the excessive oxidative stress in injured areas. In previous studies, people used antioxidative drugs such as melatonin and hormone agonists against oxidative injury in injured areas [89, 90]. We took advantage of the excellent and repeatable reversibility of cerium oxide nanoparticles and proposed a novel strategy that constructed cerium oxide nanoparticle-carried hUCMSCs. Our results indicate that the carriage of cerium oxide nanoparticles could against oxidative stress and enhance the effect of hUCMSCs. In future studies, novel bioactive hydrogel-based delivery solutions are expected to join forces with stem cell modification programs to provide a more effective intervention strategy for tendon injury. Our study has some limitations. First, given the potential ethical issue, we must explore the effects of PEG-CeONP-carrying hUCMSCs in rats, and the impact of xenogeneic transplantation should be considered. Additionally, the biological behavior of PEG-CeONP-carrying hUCMSCs in vivo should be tracked using advanced experimental methods. Finally, a synthetic scheme for producing clinical-grade PEG-CeONPs should be developed for further clinical research.

## Conclusion

This study indicates that PEG-CeONPs with repeatable reducibility were taken up by hUCMSCs by endocytosis. When exposed to excessive concentrations of H_2_O_2_, most cells carried with the PEG-CeONPs remained active to avoid senescence and apoptosis by inhibiting of ROS-induced NFκB and MAPK activation. In addition, to intracellular oxidative stress, high levels of ROS in the injured microenvironment were reduced by PEG-CeONP-carrying hUCMSCs. Intra-tendon injection of PEG-CeONP-carrying hUCMSCs facilitated tendon regeneration by preventing senescence and apoptosis. The results in our study provide a novel nongenetic strategy to improve the therapeutic potential of stem cells, which could be widely used in the transplantation of stem cells target various diseases.

## Data Availability

Data sharing is not applicable to this article as no datasets were generated or analysed during the current study.

## Abbreviations

ROS: Reactive oxygen species
CeONPs: Ceria nanoparticles
hUCMSCs: Human umbilical cord mesenchymal stem cells
MSCs: Mesenchymal stem cells
PEG-CeONPs: Methyl polyethylene glycol 2000 distearylphosphatidylethanolamine-packed CeONPs
Cy3: Cyanine3
DPPH: 2,2-Diphenyl-1-picrylhydrazyl
SOD: Superoxide dismutase
CAT: Catalase
DE: Dehydrocrenatine
FITC: Fluorescein isothiocyanate
PI: Propidium iodide
ANOVA: Analysis of variance
PTD: Patellar tendon defection

## References

[1] Lui PPY, Zhang X, Yao S, Sun H, Huang C (2022). Roles of oxidative stress in acute tendon injury and degenerative tendinopathy—a target for intervention. Int J Mol Sci.

[2] Harvey T, Flamenco S, Fan CM (2019). A Tppp3^+^Pdgfra^+^ tendon stem cell population contributes to regeneration and reveals a shared role for PDGF signalling in regeneration and fibrosis. Nat Cell Biol.

[3] Bullough R, Finnigan T, Kay A, Maffulli N, Forsyth NR (2008). Tendon repair through stem cell intervention: cellular and molecular approaches. Disabil Rehabil.

[4] Yin Z, Chen X, Chen JL, Ouyang HW (2010). Stem cells for tendon tissue engineering and regeneration. Expert Opin Biol Ther.

[5] Liu CF, Aschbacher-Smith L, Barthelery NJ, Dyment N, Butler D, Wylie C (2011). What we should know before using tissue engineering techniques to repair injured tendons: a developmental biology perspective. Tissue Eng Part B Rev.

[6] Chen HS, Chen YL, Harn HJ, Lin JS, Lin SZ (2013). Stem cell therapy for tendon injury. Cell Transpl.

[7] Lui PP, Ng SW (2013). Cell therapy for the treatment of tendinopathy—a systematic review on the pre-clinical and clinical evidence. Semin Arthritis Rheum.

[8] Caplan AI (1991). Mesenchymal stem cells. J Orthop Res.

[9] Caplan AI (2007). Adult mesenchymal stem cells for tissue engineering versus regenerative medicine. J Cell Physiol.

[10] Figueroa D, Espinosa M, Calvo R, Scheu M, Vaisman A, Gallegos M (2014). Anterior cruciate ligament regeneration using mesenchymal stem cells and collagen type I scaffold in a rabbit model. Knee Surg Sports Traumatol Arthrosc.

[11] Xie F, He J, Chen Y, Hu Z, Qin M, Hui T (2020). Multi-lineage differentiation and clinical application of stem cells from exfoliated deciduous teeth. Hum Cell.

[12] Bongso A, Fong CY (2013). The therapeutic potential, challenges and future clinical directions of stem cells from the Wharton’s jelly of the human umbilical cord. Stem Cell Rev Rep.

[13] Yan Z, Yin H, Wu J, Tian G, Li M, Liao Z (2023). Engineering exosomes by three-dimensional porous scaffold culture of human umbilical cord mesenchymal stem cells promote osteochondral repair. Mater Today Bio.

[14] Chen L, Li L, Mo Q, Zhang X, Chen C, Wu Y (2023). An injectable gelatin/sericin hydrogel loaded with human umbilical cord mesenchymal stem cells for the treatment of uterine injury. Bioeng Transl Med.

[15] Li Y, Huang J, Wang J, Xia S, Ran H, Gao L (2023). Human umbilical cord-derived mesenchymal stem cell transplantation supplemented with curcumin improves the outcomes of ischemic stroke via AKT/GSK-3beta/beta-TrCP/Nrf2 axis. J Neuroinflammation.

[16] Yea JH, Park JK, Kim IJ, Sym G, Bae TS, Jo CH (2020). Regeneration of a full-thickness defect of rotator cuff tendon with freshly thawed umbilical cord-derived mesenchymal stem cells in a rat model. Stem Cell Res Ther.

[17] Park GY, Kwon DR, Lee SC (2015). Regeneration of full-thickness rotator cuff tendon tear after ultrasound-guided injection with umbilical cord blood-derived mesenchymal stem cells in a rabbit model. Stem Cells Transl Med.

[18] Takahashi A, Okada R, Nagao K, Kawamata Y, Hanyu A, Yoshimoto S (2017). Exosomes maintain cellular homeostasis by excreting harmful DNA from cells. Nat Commun.

[19] Wang H, Nicolay BN, Chick JM, Gao X, Geng Y, Ren H (2017). The metabolic function of cyclin D3-CDK6 kinase in cancer cell survival. Nature.

[20] Murfin LC, Weber M, Park SJ, Kim WT, Lopez-Alled CM, McMullin CL (2019). Azulene-derived fluorescent probe for bioimaging: detection of reactive oxygen and nitrogen species by two-photon microscopy. J Am Chem Soc.

[21] Teo JY, Seo Y, Ko E, Leong J, Hong YT, Yang YY (2019). Surface tethering of stem cells with H_2_O_2_-responsive anti-oxidizing colloidal particles for protection against oxidation-induced death. Biomaterials.

[22] Watts AE, Yeager AE, Kopyov OV, Nixon AJ (2011). Fetal derived embryonic-like stem cells improve healing in a large animal flexor tendonitis model. Stem Cell Res Ther.

[23] Beredjiklian PK, Favata M, Cartmell JS, Flanagan CL, Crombleholme TM, Soslowsky LJ (2003). Regenerative versus reparative healing in tendon: a study of biomechanical and histological properties in fetal sheep. Ann Biomed Eng.

[24] Fernandez-Varo G, Perramon M, Carvajal S, Oro D, Casals E, Boix L (2020). Bespoken nanoceria: an effective treatment in experimental hepatocellular carcinoma. Hepatology.

[25] Yu H, Jin FY, Liu D, Shu GF, Wang XJ, Qi J (2020). ROS-responsive nano-drug delivery system combining mitochondria-targeting ceria nanoparticles with atorvastatin for acute kidney injury. Theranostics.

[26] Kim CK, Kim T, Choi IY, Soh M, Kim D, Kim YJ (2012). Ceria nanoparticles that can protect against ischemic stroke. Angew Chem Int Ed.

[27] Kwon HJ, Cha MY, Kim D, Kim DK, Soh M, Shin K (2016). Mitochondria-targeting ceria nanoparticles as antioxidants for Alzheimer’s disease. ACS Nano.

[28] Casals G, Perramon M, Casals E, Portoles I, Fernandez-Varo G, Morales-Ruiz M (2021). Cerium oxide nanoparticles: a new therapeutic tool in liver diseases. Antioxid (Basel).

[29] Luo LJ, Nguyen DD, Lai JY (2021). Harnessing the tunable cavity of nanoceria for enhancing Y-27632-mediated alleviation of ocular hypertension. Theranostics.

[30] Lee SS, Zhu HG, Contreras EQ, Prakash A, Puppala HL, Colvin VL (2012). High temperature decomposition of cerium precursors to form ceria nanocrystal libraries for biological applications. Chem Mater.

[31] Hsieh MC, Lo YS, Chuang YC, Lin CC, Ho HY, Hsieh MJ (2021). Dehydrocrenatidine extracted from *Picrasma quassioides* induces the apoptosis of nasopharyngeal carcinoma cells through the JNK and ERK signaling pathways. Oncol Rep.

[32] Machova Urdzikova L, Sedlacek R, Suchy T, Amemori T, Ruzicka J, Lesny P (2014). Human multipotent mesenchymal stem cells improve healing after collagenase tendon injury in the rat. Biomed Eng Online.

[33] Xu L, Ding L, Wang L, Cao Y, Zhu H, Lu J (2017). Umbilical cord-derived mesenchymal stem cells on scaffolds facilitate collagen degradation via upregulation of MMP-9 in rat uterine scars. Stem Cell Res Ther.

[34] Yang H, Gu S, Li J, Jin L, Xie X, Luo L (2021). Synthesis of boron carbonitride nanosheets using for delivering paclitaxel and their antitumor activity. Colloids Surf B Biointerfaces.

[35] Oda CMR, Fernandes RS, de Araujo Lopes SC, de Oliveira MC, Cardoso VN, Santos DM (2017). Synthesis, characterization and radiolabeling of polymeric nano-micelles as a platform for tumor delivering. Biomed Pharmacother.

[36] Lin Y, Xu C, Ren J, Qu X (2012). Using thermally regenerable cerium oxide nanoparticles in biocomputing to perform label-free, resettable, and colorimetric logic operations. Angew Chem Int Ed Engl.

[37] Esrafilzadeh D, Zavabeti A, Jalili R, Atkin P, Choi J, Carey BJ (2019). Room temperature CO_2_ reduction to solid carbon species on liquid metals featuring atomically thin ceria interfaces. Nat Commun.

[38] Kraft VAN, Bezjian CT, Pfeiffer S, Ringelstetter L, Muller C, Zandkarimi F (2020). GTP cyclohydrolase 1/tetrahydrobiopterin counteract ferroptosis through lipid remodeling. ACS Cent Sci.

[39] Zhang LS, Davies SS (2016). Microbial metabolism of dietary components to bioactive metabolites: opportunities for new therapeutic interventions. Genome Med.

[40] Griendling KK, Touyz RM, Zweier JL, Dikalov S, Chilian W, Chen YR (2016). Measurement of reactive oxygen species, reactive nitrogen species, and Redox-dependent signaling in the cardiovascular system: a scientific statement from the American Heart Association. Circ Res.

[41] Mohamed BA, Schnelle M, Khadjeh S, Lbik D, Herwig M, Linke WA (2016). Molecular and structural transition mechanisms in long-term volume overload. Eur J Heart Fail.

[42] Kadlec AO, Beyer AM, Ait-Aissa K, Gutterman DD (2016). Mitochondrial signaling in the vascular endothelium: beyond reactive oxygen species. Basic Res Cardiol.

[43] Khalafalla FG, Greene S, Khan H, Ilves K, Monsanto MM, Alvarez R (2017). P2Y_2_ nucleotide receptor prompts human cardiac progenitor cell activation by modulating Hippo signaling. Circ Res.

[44] Kwon E, Todorova K, Wang J, Horos R, Lee KK, Neel VA (2018). The RNA-binding protein YBX1 regulates epidermal progenitors at a posttranscriptional level. Nat Commun.

[45] Galluzzi L, Vitale I, Aaronson SA, Abrams JM, Adam D, Agostinis P (2018). Molecular mechanisms of cell death: recommendations of the Nomenclature Committee on cell death 2018. Cell Death Differ.

[46] Fuhrmann-Stroissnigg H, Ling YY, Zhao J, McGowan SJ, Zhu Y, Brooks RW (2017). Identification of HSP90 inhibitors as a novel class of senolytics. Nat Commun.

[47] Paithankar JG, Saini S, Dwivedi S, Sharma A, Chowdhuri DK (2021). Heavy metal associated health hazards: an interplay of oxidative stress and signal transduction. Chemosphere.

[48] Chong SJF, Lai JXH, Eu JQ, Bellot GL, Pervaiz S (2018). Reactive oxygen species and oncoprotein signaling—a dangerous liaison. Antioxid Redox Signal.

[49] McGovern A, Schoenfelder S, Martin P, Massey J, Duffus K, Plant D (2016). Capture Hi-C identifies a novel causal gene, IL20RA, in the pan-autoimmune genetic susceptibility region 6q23. Genome Biol.

[50] Ho HY, Lin CC, Chuang YC, Lo YS, Hsieh MJ, Chen MK (2021). Apoptotic effects of dehydrocrenatidine via JNK and ERK pathway regulation in oral squamous cell carcinoma. Biomed Pharmacother.

[51] Elliott DH (1965). Structure and function of mammalian tendon. Biol Rev Camb Philos Soc.

[52] Kyurkchiev D, Bochev I, Ivanova-Todorova E, Mourdjeva M, Oreshkova T, Belemezova K (2014). Secretion of immunoregulatory cytokines by mesenchymal stem cells. World J Stem Cells.

[53] Ma S, Xie N, Li W, Yuan B, Shi Y, Wang Y (2014). Immunobiology of mesenchymal stem cells. Cell Death Differ.

[54] Baraniak PR, McDevitt TC (2010). Stem cell paracrine actions and tissue regeneration. Regen Med.

[55] Hammond OS, Edler KJ, Bowron DT, Torrente-Murciano L (2017). Deep eutectic-solvothermal synthesis of nanostructured ceria. Nat Commun.

[56] Esch F, Fabris S, Zhou L, Montini T, Africh C, Fornasiero P (2005). Electron localization determines defect formation on ceria substrates. Science.

[57] Heckert EG, Karakoti AS, Seal S, Self WT (2008). The role of cerium redox state in the SOD mimetic activity of nanoceria. Biomaterials.

[58] Pirmohamed T, Dowding JM, Singh S, Wasserman B, Heckert E, Karakoti AS (2010). Nanoceria exhibit redox state-dependent catalase mimetic activity. Chem Commun (Camb).

[59] Cafun JD, Kvashnina KO, Casals E, Puntes VF, Glatzel P (2013). Absence of Ce^3+^ sites in chemically active colloidal ceria nanoparticles. ACS Nano.

[60] Celardo I, Pedersen JZ, Traversa E, Ghibelli L (2011). Pharmacological potential of cerium oxide nanoparticles. Nanoscale.

[61] Liu J, Zhu P, Song P, Xiong W, Chen H, Peng W (2015). Pretreatment of adipose derived stem cells with Curcumin facilitates myocardial recovery via antiapoptosis and angiogenesis. Stem Cells Int.

[62] Liu J, Wang H, Wang Y, Yin Y, Wang L, Liu Z (2014). Exendin-4 pretreated adipose derived stem cells are resistant to oxidative stress and improve cardiac performance via enhanced adhesion in the infarcted heart. PLoS ONE.

[63] Zhang Z, Zhang M, Sun Y, Li M, Chang C, Liu W (2023). Effects of adipose derived stem cells pretreated with resveratrol on sciatic nerve regeneration in rats. Sci Rep.

[64] Kim YG, Lee Y, Lee N, Soh M, Kim D, Hyeon T. Ceria-based therapeutic antioxidants for biomedical applications. Adv Mater. 2023;15:e2210819. 10.1002/adma.202210819 36793245

[65] Wang H, Agarwal P, Zhao G, Ji G, Jewell CM, Fisher JP (2018). Overcoming ovarian cancer drug resistance with a cold responsive nanomaterial. ACS Cent Sci.

[66] Lin W, Huang YW, Zhou XD, Ma Y (2006). Toxicity of cerium oxide nanoparticles in human lung cancer cells. Int J Toxicol.

[67] De Marzi L, Monaco A, De Lapuente J, Ramos D, Borras M, Di Gioacchino M (2013). Cytotoxicity and genotoxicity of ceria nanoparticles on different cell lines in vitro. Int J Mol Sci.

[68] Boada-Romero E, Martinez J, Heckmann BL, Green DR (2020). The clearance of dead cells by efferocytosis. Nat Rev Mol Cell Biol.

[69] Li K, Deng Y, Deng G, Chen P, Wang Y, Wu H (2020). High cholesterol induces apoptosis and autophagy through the ROS-activated AKT/FOXO1 pathway in tendon-derived stem cells. Stem Cell Res Ther.

[70] Jung SH, Lee M, Park HA, Lee HC, Kang D, Hwang HJ (2019). Integrin alpha6beta4-Src-AKT signaling induces cellular senescence by counteracting apoptosis in irradiated tumor cells and tissues. Cell Death Differ.

[71] Ogrodnik M, Zhu Y, Langhi LGP, Tchkonia T, Kruger P, Fielder E (2019). Obesity-induced cellular senescence drives anxiety and impairs neurogenesis. Cell Metab.

[72] Xu M, Pirtskhalava T, Farr JN, Weigand BM, Palmer AK, Weivoda MM (2018). Senolytics improve physical function and increase lifespan in old age. Nat Med.

[73] Kovatcheva M, Liao W, Klein ME, Robine N, Geiger H, Crago AM (2017). ATRX is a regulator of therapy induced senescence in human cells. Nat Commun.

[74] Zhang X, Lin YC, Rui YF, Xu HL, Chen H, Wang C (2016). Therapeutic roles of tendon stem/progenitor cells in tendinopathy. Stem Cells Int..

[75] Salminen A, Huuskonen J, Ojala J, Kauppinen A, Kaarniranta K, Suuronen T (2008). Activation of innate immunity system during aging: NF-kB signaling is the molecular culprit of inflamm-aging. Ageing Res Rev.

[76] Cilloni D, Martinelli G, Messa F, Baccarani M, Saglio G (2007). Nuclear factor kB as a target for new drug development in myeloid malignancies. Haematologica.

[77] Morgan MJ, Liu ZG (2011). Crosstalk of reactive oxygen species and NF-kappaB signaling. Cell Res.

[78] Collins KH, Herzog W, MacDonald GZ, Reimer RA, Rios JL, Smith IC (2018). Obesity, metabolic syndrome, and musculoskeletal disease: common inflammatory pathways suggest a central role for loss of muscle integrity. Front Physiol.

[79] Kamiyama M, Shirai T, Tamura S, Suzuki-Inoue K, Ehata S, Takahashi K (2017). ASK1 facilitates tumor metastasis through phosphorylation of an ADP receptor P2Y_12_ in platelets. Cell Death Differ.

[80] Choi SA, Kim YH, Park YH, Yang HJ, Jeong PS, Cha JJ (2019). Novel crosstalk between Vps26a and Nox4 signaling during neurogenesis. Cell Death Differ.

[81] Schinzel RT, Higuchi-Sanabria R, Shalem O, Moehle EA, Webster BM, Joe L (2019). The hyaluronidase, TMEM2, promotes ER homeostasis and longevity independent of the UPR(ER). Cell.

[82] Tran AT, Chapman EM, Flamand MN, Yu B, Krempel SJ, Duchaine TF (2019). MiR-35 buffers apoptosis thresholds in the *C. elegans* germline by antagonizing both MAPK and core apoptosis pathways. Cell Death Differ.

[83] Norelli JB, Plaza DP, Stal DN, Varghese AM, Liang H, Grande DA (2018). Tenogenically differentiated adipose-derived stem cells are effective in Achilles tendon repair in vivo. J Tissue Eng.

[84] Liu H, Zhu S, Zhang C, Lu P, Hu J, Yin Z (2014). Crucial transcription factors in tendon development and differentiation: their potential for tendon regeneration. Cell Tissue Res.

[85] Hou J, Yang R, Vuong I, Li F, Kong J, Mao HQ (2021). Biomaterials strategies to balance inflammation and tenogenesis for tendon repair. Acta Biomater.

[86] Wang Z, Lee WJ, Koh BTH, Hong M, Wang W, Lim PN (2018). Functional regeneration of tendons using scaffolds with physical anisotropy engineered via microarchitectural manipulation. Sci Adv.

[87] Ren Z, Duan Z, Zhang Z, Fu R, Zhu C, Fan D (2023). Instantaneous self-healing and strongly adhesive self-adaptive hyaluronic acid-based hydrogel for controlled drug release to promote tendon wound healing. Int J Biol Macromol.

[88] Ji W, Han F, Feng X, Shi L, Ma H, Lu Y (2023). Cocktail-like gradient gelatin/hyaluronic acid bioimplant for enhancing tendon-bone healing in fatty-infiltrated rotator cuff injury models. Int J Biol Macromol.

[89] Yao Z, Qian Y, Jin Y, Wang S, Li J, Yuan WE (2022). Biomimetic multilayer polycaprolactone/sodium alginate hydrogel scaffolds loaded with melatonin facilitate tendon regeneration. Carbohydr Polym.

[90] Shen H, Cheng L, Zheng Q, Liu W, Wang Y (2022). Scavenging of reactive oxygen species can adjust the differentiation of tendon stem cells and progenitor cells and prevent ectopic calcification in tendinopathy. Acta Biomater.

